# Exercise Training Attenuates Hypertension via Suppressing ROS/MAPK/NF-κB/AT-1R Pathway in the Hypothalamic Paraventricular Nucleus

**DOI:** 10.3390/nu14193968

**Published:** 2022-09-24

**Authors:** Jie Qi, Rui-Juan Li, Li-Yan Fu, Kai-Li Liu, Jin-An Qiao, Yu Yang, Xiao-Jing Yu, Jia-Yue Yu, Ying Li, Hong Tan, Yu-Ming Kang

**Affiliations:** 1 Department of Physiology and Pathophysiology, School of Basic Medical Sciences, Xi’an Jiaotong University Health Science Center, Xi’an Jiaotong University, Xi’an 710061, China; 2 Institute of Cardiovascular Sciences, Translational Medicine Institute, Xi’an Jiaotong University Health Science Center, Xi’an Jiaotong University, Xi’an 710061, China; 3 Key Laboratory of Environment and Genes Related to Diseases, Ministry of Education, Xi’an Jiaotong University, Xi’an 710061, China; 4 Department of Infectious Diseases, The Second Affiliated Hospital, Air Force Military Medical University, Xi’an 710038, China; 5 Institute of Pediatric Diseases, Xi’an Children’s Hospital, Xi’an 710002, China; 6 College of Life Sciences, Northwest University, Xi’an 710069, China

**Keywords:** exercise training, paraventricular nucleus, hypertension, angiotensin II type 1 receptor, MAPK

## Abstract

Background: Aerobic exercise training (ExT) is beneficial for hypertension, however, its central mechanisms in improving hypertension remain unclear. Since the importance of the up-regulation of angiotensin II type 1 receptor (AT-1R) in the paraventricular nucleus (PVN) of the hypothalamic in sympathoexcitation and hypertension has been shown, we testified the hypothesis that aerobic ExT decreases blood pressure in hypertensive rats by down-regulating the AT-1R through reactive oxygen species (ROS)/mitogen-activated protein kinase (MAPK)/nuclear factors κB (NF-κB) pathway within the PVN. Methods: Forty-eight male Sprague-Dawley (SD) rats were assigned to the following groups: sham operation (SHAM) + kept sedentary (Sed), SHAM + exercise training (ExT), two kidney-one clamp (2K1C) + Sed, and 2K1C + ExT groups. Results: The 2K1C + Sed hypertensive rats showed higher systolic blood pressure (SBP), upregulated ROS, phosphorylated (p-) p44/42 MAPK, p-p38 MAPK, NF-κB p65 activity, and AT-1R expression in the PVN, and increased circulating norepinephrine (NE) than those of SHAM rats. After eight weeks of aerobic ExT, the 2K1C + ExT hypertensive rats showed attenuated NE and SBP levels, suppressed NF-κB p65 activity, and reduced expression of ROS, p-p44/42 MAPK, p-p38 MAPK, and AT-1R in the PVN, relatively to the 2K1C + Sed group. Conclusions: These data are suggestive of beneficial effects of aerobic ExT in decreasing SBP in hypertensive rats, via down-regulating the ROS/MAPK/NF-κB pathway that targets AT-1R in the PVN, and eventually ameliorating 2K1C-induced hypertension.

## 1. Introduction

Hypertension is a syndrome with elevated blood pressure as the primary clinical manifestation. A new study published in the Lancet journal reported that the number of hypertensive patients aged from 30 to 79 almost doubled in the world, from 650 million to 1.28 billion, during the 30-year period 1990–2019 [1]. According to the “2018 Chinese guidelines for the management of hypertension”, the occurrence of hypertension keeps increasing and currently is 27.9% among residents aged 18 and over in China. These epidemiology studies demonstrate that hypertension has become a critical public health issue. Numerous studies have shown that the autonomic nervous system is dysfunctional in hypertension, that is, increased sympathetic nerve activity (SNA) and relatively decreased vagal tone [2,3]. In recent years, clinical studies and animal experiments have confirmed that renal sympathetic denervation by radiofrequency ablation can effectively treat refractory hypertension [4,5]. It shows that the enhanced SNA plays an important role in the onset and progression of hypertension [6].

Accumulating evidence demonstrates that hypothalamic paraventricular nucleus (PVN) is the major brain nucleus containing sympathetic preganglionic neurons [7]. The sympathetic preganglionic neurons are directly innervated by PVN and participate in the modulation of SNA [8]. Pathological activation of these sympathetic preganglionic neurons in the PVN leads to enhanced SNA during the onset and progression of hypertension [9,10].

Recent studies demonstrated the existence as well as the importance of renin-angiotensin system (RAS) in the humoral system [11]. RAS including angiotensin II (ANG II) type 1 receptor (AT-1R) also presents in the brain tissue [12,13]. Recent findings suggest that AT1-R within PVN is upregulated in hypertensive and heart failure rats [14,15,16]. It was reported that the central blockade of AT-1R decreases renal sympathetic nerve activity (RSNA) and blood pressure in ANG II or high salt induced hypertensive rats [10,17,18]. Our previous study found that aerobic exercise training (ExT) can reduce the expression of AT1-R in the PVN, reduce SNA and thereby effectively control hypertension [19], but the central mechanism is not clear.

Previous studies have reported reactive oxygen species (ROS) production in the PVN, which induces abnormal sympathetic activity in the hypertensive rats [20,21]. ROS within the PVN was shown to play important roles in regulating RSNA in the ANG II or high salt-induced hypertension animal models [22,23]. ROS can further activate a variety of downstream signaling molecules including protein kinases, of which the most important one is the mitogen-activated protein kinase (MAPK) family [24]. The p44/42 MAPK, c-Jun N-terminal kinase (JNK) and p38 MAPK are the major members of the MAPK family. Robert B Felder’s research team found that the PVN level of phosphorylated (p-) p44/42 MAPK and p-p38 MAPK was increased, and the RSNA was enhanced in the heart failure rats; however, JNK in the PVN had little effect on SNA [25,26]. The results suggest that, in the PVN of heart failure model, there is a close correlation between sympathetic excitation and MAPK signal. Thus, in the current investigation, the effect of aerobic ExT mediated by p44/42 MAPK and p38 MAPK signaling in the PVN was emphasized in the hypertensive rat model. Nuclear factor κB (NF-κB), a well-known transcriptional factor, participates in the regulation of early immune responses related to body defense function and inflammatory response [27]. Activated NF-κB can increase the synthesis of RAS in the PVN, and thus cause sympathetic excitation and hypertensive response [17].

To sum up, in this study, to expose underlying mechanisms leading to down-regulation of AT-1R in PVN following ExT, the hypothesis that ExT suppresses the ROS/MAPK/NF-κB/AT-1R pathway within the PVN and attenuates hypertension progression was tested.

## 2. Materials and Methods

### 2.1. Animals

Healthy male Sprague-Dawley (SD) rats (5–6 weeks old) used in the experiment were maintained in the laboratory animal center of Xi’an Jiaotong University. The Animal Care and Use Committee of the same institution approved the animal protocols (No. 2021-202). They were firstly housed for a week of acclimatization. The hypertensive rat model was prepared by the two kidney-one clamp (2K1C) operation. Eating and drinking were forbidden for 12 h and for 4 h before surgery. Experiments were carried out in agreement with the National Institutes of Health guide for the care and use of Laboratory Animals (NIH Publication No. 85-23, revised 1996).

### 2.2. Exercise Training Protocols

**Figure 1:**
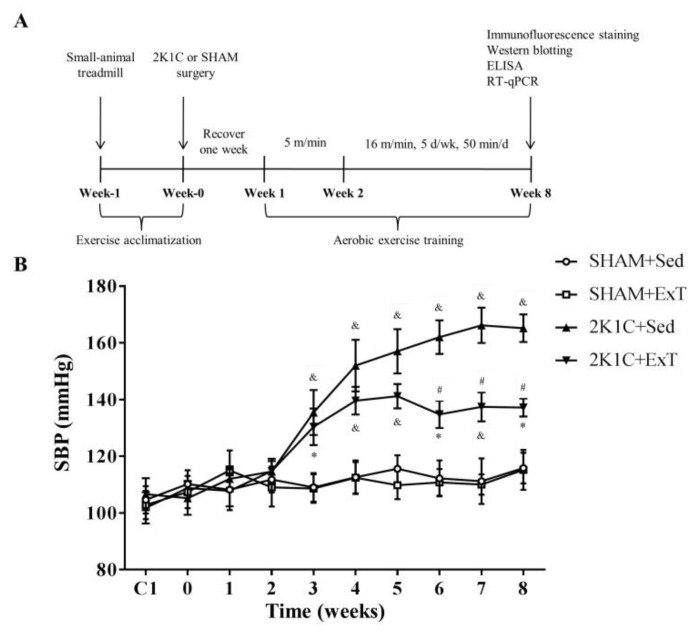
The schematic diagram of blood pressure curves of the four experimental groups. (**A**) Experimental design. (**B**) Aerobic ExT attenuated SBP from the 6th week to the end of the experiment. (*n* = 5 rats, * *p* < 0.05 or ^&^ *p* < 0.01 compared with the SHAM + Sed group or the SHAM + ExT group; ^#^ *p* < 0.01 relatively to 2K1C + Sed group).

All rats were pre-adapted to the training program in the small animal treadmill (FT-200, Chengdu Techman Software Co., Ltd., Chengdu, China) for 5 days. Rats that could not adhere to the aerobic exercise training [28,29,30] (50 min/day, 5 days/week, 50–60% of maximal exercise capacity) were removed, and the rats that met the aerobic exercise training were retained and screened. The eligible rats were randomly divided into an aerobic exercise training group (ExT) or kept sedentary group (Sed). Each group was randomly split into two: sham operation (SHAM) group or two kidney-one clamp (2K1C) operation group, resulting in four groups namely SHAM + Sed, SHAM + ExT, 2K1C + Sed, and 2K1C + ExT. Briefly, the SD rats were anesthetized with isoflurane (500–700 mL/min). Through a right abdominal incision, the right renal artery was exposed for the acupuncture needle placement. The right renal artery and acupuncture needle were ligated with silk thread (2-0), and then the acupuncture needle was drawn out to induce hypertension [31]. A sham procedure was carried out as control. All rats recovered for one week after surgery. From the second week after the operation, the rats of the ExT group started running at 5.0 m/min for 50 min over 3–5 days. Then the speed gradually increased to 16.0 m/min from the three week till the eighth week (Figure 1A).

### 2.3. Recording of Blood Pressure

SBP of all rats were recorded using the tail-cuff method daily before the operation as previously described [32]. During the experiment, the SBP was measured once per week.

### 2.4. Renal Sympathetic Nerve Recordings

RSNA parameter measurements were conducted as described earlier [33]. Briefly, under general anesthesia with a mixture of ketamine (80 mg/kg) and xylazine (10 mg/kg) (intraperitoneal), rats underwent a retroperitoneal laparotomy and the left renal nerves were identified for RSNA recording.

### 2.5. Collection of Brain Tissue Samples and Blood

Animals were anesthetized with isoflurane at the end of 8th week, plasma specimens and brain tissue were gathered and stored at −80 °C as previously described [34].

### 2.6. Immunofluorescence Staining

The intact brain was removed from the skull and fixed in 4% paraformaldehyde for 12 h and then transferred to 30% sucrose solution prepared with 0.01 M phosphate buffer for 3 days. The fixed brain tissue was embedded by OCT, then quickly frozen, and then frozen sectioned with a thickness of 18 µm. As mentioned previously, immunofluorescence studies were performed in floating sections [35]. The primary antibodies for p-p44/42 MAPK (Thr202/Tyr204, 1:200 dilution) and p-p38 MAPK (Thr180/Tyr182, 1:200 dilution) were bought from CST, AT-1R (ab124505, 1:20 dilution) was bought from Abcam. Dihydroethidium (DHE, Molecular Probes) was to check ROS generation.

### 2.7. Western Blotting

Total protein samples were electrophoresed on a polyacrylamide gel for 1.5 h then transferred to PVDF membranes. After transfer, the membranes were blocked in 5% BSA solution, then incubated with primary antibody overnight at 4 °C. The membranes were washed and incubated with secondary antibody (room temperature, 1 h) [36]. The primary antibodies for p-p44/42 MAPK (#9101, 1:1000 dilution), p44/42 MAPK (#4695, 1:1000 dilution), p-p38 MAPK (#4511, 1:1000 dilution) and p38 MAPK (#9212, 1:1000 dilution, CST, Danvers, MA, USA), AT-1R (ab124505, 1:1000 dilution, Abcam, Cambridge, UK) were used.

### 2.8. ELISA Studies

Circulating norepinephrine (NE) and NF-κB p65 activity in the PVN were measured using ELISA kits, as previously described [22].

### 2.9. Quantitative Real-Time PCR (RT-qPCR)

AT-1R mRNA in PVN was quantified through RT-qPCR and the list of primer sequences is presented in Table 1 [37]. RT-qPCR was performed as noted earlier [34]. GAPDH level is standardized by the gene expression level of AT-1R mRNA.

### 2.10. Statistical Analysis

Results were presented as mean ± SEM and statistical significance was considered for *p* < 0.05. Data were analyzed by two-way ANOVA followed by a post-hoc Tukey test, and blood pressure data were analyzed by repeated measures ANOVA.

## 3. Results

### 3.1. Blood Pressure

The blood pressure of the tail arteries of the SD rats in the four groups was measured at a fixed time point every week. The basal SBP in each group was similar. Figure 1B shows significant increases in SBP of the 2K1C rats starting from the third week to the end of the study (compared to the sedentary rats in SHAM group, * *p* < 0.05 ^&^ *p* < 0.01). Aerobic ExT significantly attenuated SBP starting from the 6th week in 2K1C + ExT (contrasted to 2K1C + Sed group, ^#^ *p* < 0.01). Our results showed that aerobic ExT attenuated blood pressure in hypertensive rats.

### 3.2. Renal Sympathetic Nerve Activity

**Figure 2:**
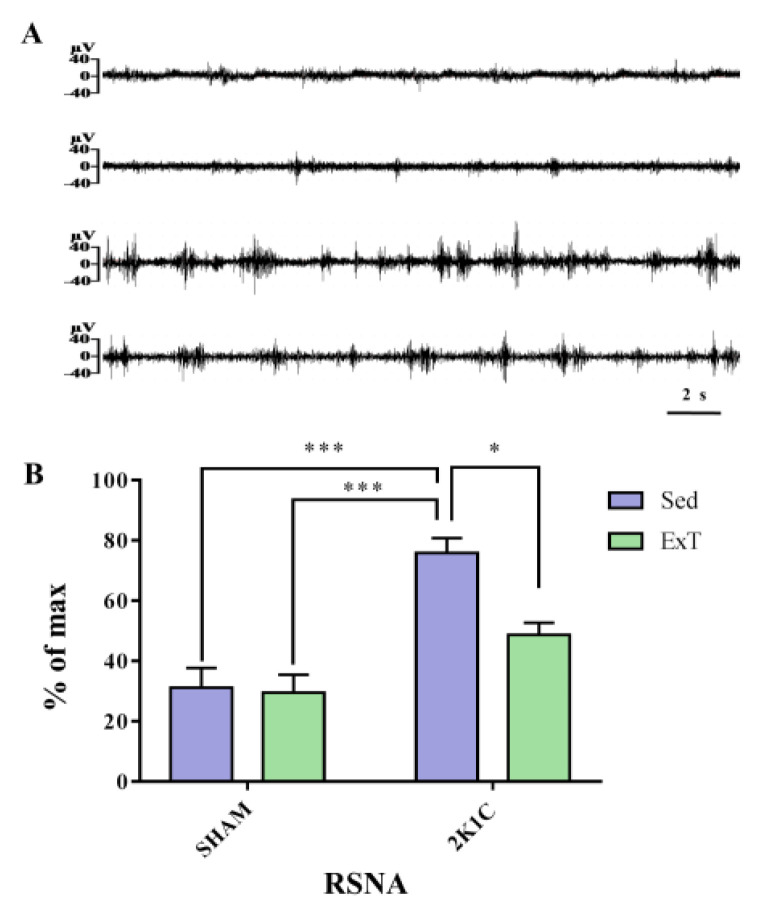
The effect of eight-week of aerobic ExT on RSNA. (**A**) The schematic diagram of RSNA. (**B**) Statistical analysis of the level of RSNA. * *p* < 0.05, *** *p* < 0.001, *n* = 4.

Recent studies have found that the kidneys are only innervated by the sympathetic nerves, but not by vagus nerves [38]. The renal sympathetic nerve plays a vital role in the regulation of systemic autonomic balance. Excessive renal sympathetic nerve activation can promote the progression of angiocardiopathy like hypertension, therefore we measured the level of RSNA. Figure 2A,B show that sedentary rats in the 2K1C renovascular hypertension group exhibited higher RSNA than the sedentary rats in SHAM group (*p* < 0.001). With eight-week aerobic exercise training treatment, RSNA was significantly reduced comparatively to sedentary 2K1C animal group (*p* < 0.05). These results revealed that RSNA was negatively regulated by aerobic ExT in 2K1C rats.

### 3.3. Plasma NE

**Figure 3:**
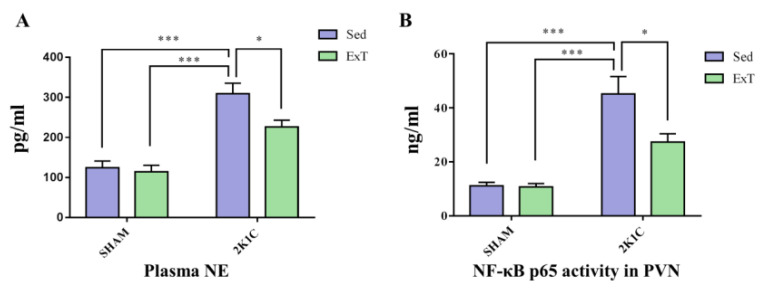
The effect of eight weeks of aerobic ExT treatment on the level of circulating NE and on the NF-κB activation in the PVN in the four groups. (**A**) Statistical analysis of NE. (**B**) Statistical analysis of NF-κB p65 activity. * *p* < 0.05, *** *p* < 0.001, *n* = 4–5.

The plasma NE level indicates the degree of the SNA. Figure 3A shows that compared to the sedentary rats in SHAM group, sedentary rats in 2K1C renovascular hypertension group had higher plasma NE (*p* < 0.001). Eight weeks of aerobic exercise training decreased the level of circulating NE in 2K1C + ExT group as compared to the 2K1C + Sed rats (*p* < 0.05). These results demonstrated that aerobic ExT decreased plasma NE level in 2K1C rats.

### 3.4. DHE Expression in the PVN

**Figure 4:**
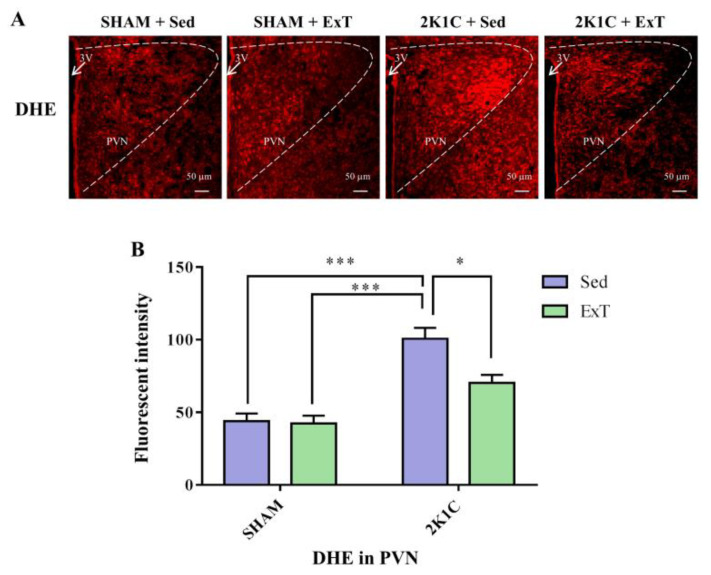
The effect of eight weeks of aerobic ExT treatment on ROS expression. (**A**) Representative images of DHE staining. (**B**) Densitometric analysis of DHE. 3V: third ventricle. * *p* < 0.05, *** *p* < 0.001, *n* = 4.

To examine the ROS production in PVN, DHE staining was performed. The fluorescent intensity of DHE was quantified by NIH Image J2x software (Washington, DC, USA). Figure 4A,B show that compared with SHAM rats, 2K1C + Sed rats exhibited an increased ROS level (*p* < 0.001). Eight weeks of aerobic exercise training attenuated the ROS compared with the sedentary rats in 2K1C group rats (*p* < 0.05). These data therefore revealed aerobic ExT reduced ROS production in 2K1C hypertensive rats.

### 3.5. P-p44/42 MAPK Expression in the PVN

**Figure 5:**
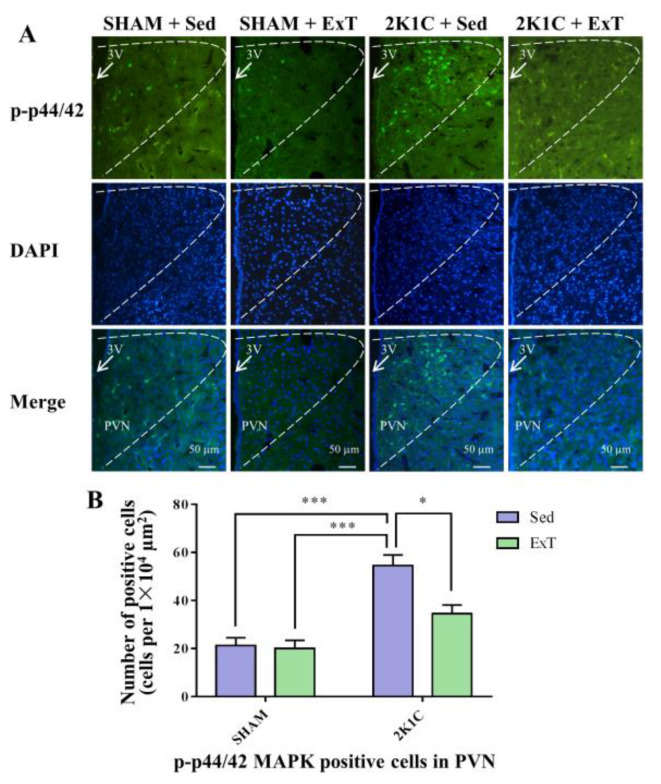
Eight weeks of aerobic ExT attenuated PVN level of p-p44/42 MAPK immunoreactivity in 2K1C group. (**A**) Representative immunofluorescence staining of p-p44/42 MAPK. (**B**) Densitometric analysis of immunofluorescence staining of p-p44/42 MAPK. 3V: third ventricle. * *p* < 0.05, *** *p* < 0.001, *n* = 4.

**Figure 6:**
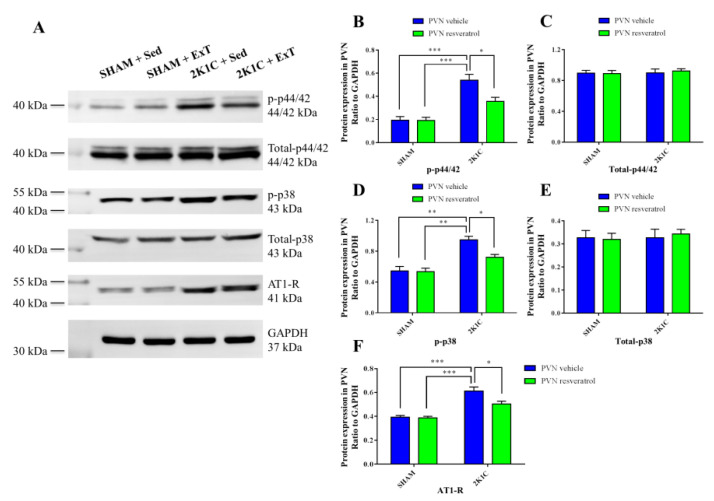
Eight weeks of aerobic ExT attenuated PVN protein expression for p-p44/42 MAPK, p-p38 MAPK, and AT-1R in 2K1C group. (**A**) Representative immunoblots of p-p44/42 MAPK, total-p44/42 MAPK, p-p38 MAPK, total-p38 MAPK and AT-1R. (**B**,**C**) Densitometry of protein expressions of p-p44/42 MAPK and total-p44/42 MAPK. (**D**,**E**) Densitometry of protein expressions of p-p38 MAPK and total-p38 MAPK. (**F**) Densitometry of protein expressions of AT-1R. * *p* < 0.05, ** *p* < 0.01, *** *p* < 0.001, *n* = 3.

We then sought to understand the underlying signaling pathway mediating effects of ExT on hypertension in PVN. Figure 5A,B, Figure 6B and Figure S1 show that sedentary rats in the 2K1C renovascular hypertension group had enhanced p-p44/42 MAPK protein expression compared to that in sedentary rats in SHAM group (*p* < 0.001). With 8-week ExT, the increased p-p44/42 MAPK activation in 2K1C + Sed rats was attenuated (Figure 5A,B, Figure 6B and Figure S1, *p* < 0.05). No statistical difference in p44/42 MAPK expression among the four groups (Figure 6C, *p* > 0.05) was detected. We observed that aerobic ExT decreased the p-p44/42 MAPK level in hypertensive rats.

### 3.6. P-p38 MAPK Expression in the PVN

**Figure 7:**
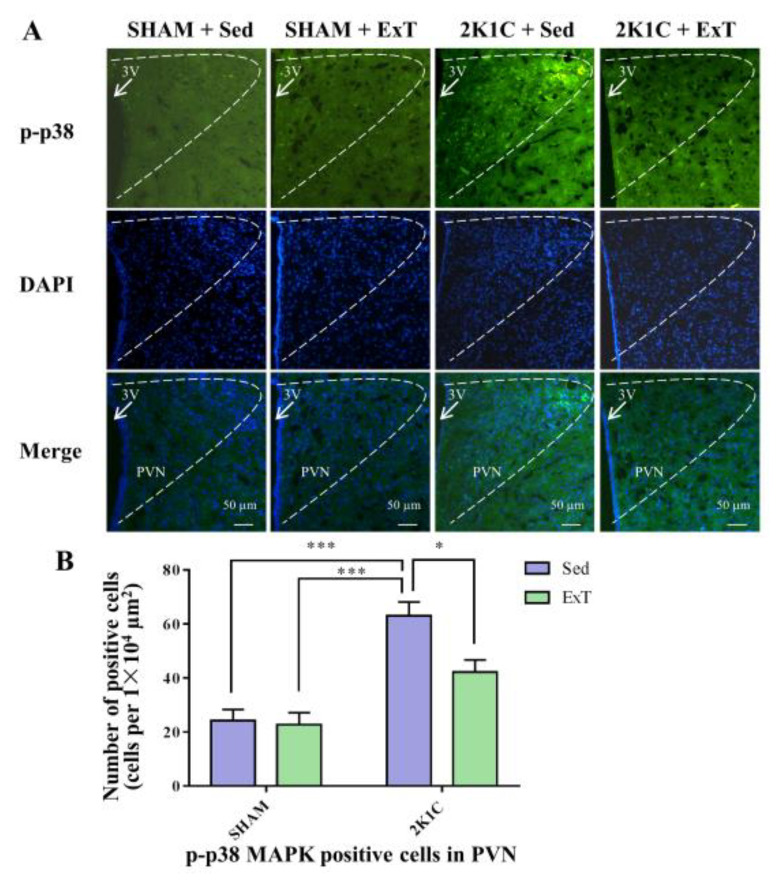
Eight weeks of aerobic ExT attenuated PVN level of p-p38 MAPK immunoreactivity in 2K1C group. (**A**) Representative immunofluorescence staining of p-p38 MAPK. (**B**) Densitometric analysis of immunofluorescence staining of p-p38 MAPK. 3V: third ventricle. * *p* < 0.05, *** *p* < 0.001, *n* = 4.

Figure 6D, Figure 7A,B and Figure S2 show that sedentary rats in the 2K1C renovascular hypertension group had enhanced p-p38 MAPK protein expression in comparison with the sedentary rats in SHAM group (*p* < 0.01). With 8-week ExT, the increased p-p38 MAPK activation in 2K1C + Sed rats was attenuated (Figure 6D, Figure 7A,B and Figure S2, *p* < 0.05). No statistical difference in p38 MAPK expression among the four groups (Figure 6E, *p* > 0.05) was detected. These results indicated decreased p-p38 MAPK protein expression in hypertensive rats.

### 3.7. NF-κB Activity in the PVN

Figure 3B shows that NF-κB p65 expression in sedentary rats of the 2K1C renovascular hypertension group was higher than that in SHAM group (*p* < 0.001). Eight weeks of aerobic exercise training attenuated the increase in 2K1C + ExT group as contrasted with the 2K1C + Sed group (*p* < 0.05). We observed that ExT inhibited the activity of NF-κB in hypertensive rats.

### 3.8. AT-1R Expression in the PVN

**Figure 8:**
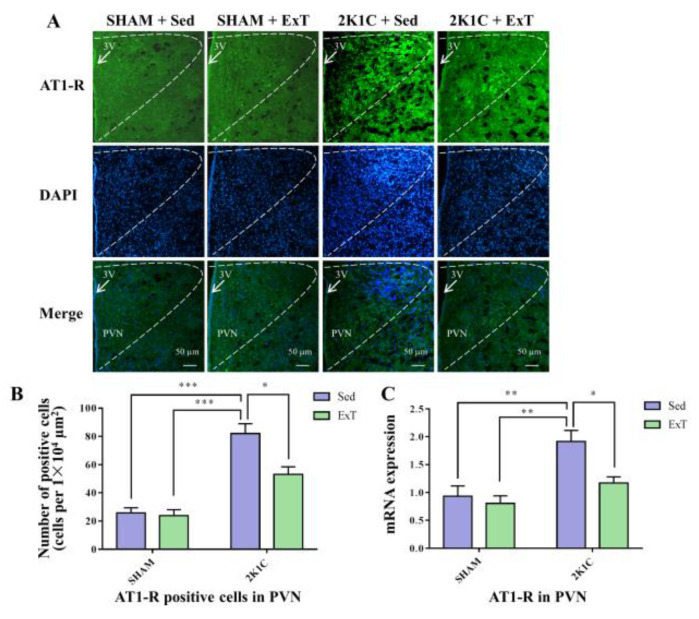
Eight weeks of aerobic ExT attenuated AT-1R immunoreactivity in the PVN of the 2K1C group. (**A**) Representative images of immunofluorescence staining of AT-1R. (**B**) Densitometric analysis of immunoreactivity for AT-1R. (**C**) Densitometric analysis of RT-qPCR of AT-1R. 3V: third ventricle. * *p* < 0.05, ** *p* < 0.01, *** *p* < 0.001, *n* = 4.

Figure 8A–C, Figure 6F and Figure S3 show that sedentary rats in the 2K1C renovascular hypertension group had enhanced AT-1R positive cells (Figure 8A,B, *p* < 0.001) and protein expression (Figure 6F and Figure S3, *p* < 0.001), and mRNA expression (Figure 8C, *p* < 0.01) in comparison with the sedentary SHAM rats. With 8-week ExT, the increased AT-1R positive cells (Figure 8A,B, *p* < 0.05), protein expression (Figure 6F and Figure S3, *p* < 0.05) and mRNA expression (Figure 8C, *p* < 0.05) in 2K1C + Sed rats were attenuated. These data together supported that aerobic ExT inhibited AT-1R expression in 2K1C rats.

## 4. Discussion

The new findings are as follows: (i) The ROS/MAPK/NF-κB/AT-1R pathway was activated within the PVN during hypertension progression, (ii) long-term aerobic ExT significantly suppressed the ROS/MAPK/NF-κB/AT-1R pathway in the PVN, (iii) long-term aerobic ExT weakened RSNA and SBP in 2K1C hypertension. These findings revealed that aerobic ExT attenuated blood pressure likely via the ROS/MAPK/NF-κB/AT-1R pathway in PVN during hypertension.

In this study, in comparison with the control group, rats in 2K1C + ExT group showed significant decreases of SBP during the sixth to eighth weeks of the experimental period. The present study demonstrated that long-term aerobic ExT could attenuate 2K1C-induced hypertension and delay the progression of high blood pressure. Similar effects from other research groups have also been reported [39,40]. Aerobic ExT has been recognized as the basis treatment of hypertension [41].

PVN is considered as a pivotal nucleus in the brain that functions in balancing SNA and blood pressure [42]. Lots of findings have reported that the increased ROS generation, and NF-κB activation in the PVN contribute to elevated SNA in ANG II- and high salt-induced hypertensive rats [22,43,44,45]. Similarly, the outcomes of this study suggest that compared with SHAM rats, 2K1C induced hypertensive rats had enhanced ROS generation, NF-κB p65 activation and AT-1R expression within the PVN. In our experimental setting, eight weeks of aerobic ExT significantly decreased the expressions of ROS and AT-1R, as well as weakened NF-κB activation in the PVN.

It is also well established that enhanced ROS generation is associated with NF-κB activation in PVN [46,47]. Recently, it was shown that ROS is sufficient to activate NF-κB in various modes of fibroblast senescence [48]. Previous studies have already revealed that ROS promote NF-κB activation in the PVN, further increasing ROS generation in hypertensive rats [49,50]. In addition, chronic NF-κB blockade in PVN significantly attenuates RSNA in heart failure rats [51], inhibits ROS expression, and reduces blood pressure in hypertensive rats [46]. There may be a mutually promoting relationship between ROS and NF-κB in the PVN in hypertension. In addition, activated NF-κB in PVN is a master regulator of AT-1R up-regulation, and contributes to increased sympathetic activity in hypertensive [49] as well as heart failure rats [52].

MAPK, including p44/42 MAPK, JNK, and p38 MAPK as the major family members, plays pivotal roles in heart failure [53] and hypertension [54]. Recently, it was reported that phosphorylation of the MAPK within the PVN contributes to excessive RSNA, and the p44/42 MAPK inhibitor, PD-98059, micro-injected in the PVN significantly reduces RSNA in both heart failure [26] and ANG II-induced hypertensive rats [54]. This suggests that p44/42 MAPK signaling in PVN plays a key role in sympatho-excitation in the pathophysiology of heart failure and hypertension. Researchers also found p-p44/42 MAPK activation and AT1-R expression in the PVN were substantially elevated in hypertensive model in rats. Injection of PD-98059 into PVN reduced the expression of p-p44/42 MAPK and AT1-R in the PVN and consequently decreased blood pressure [55]. Therefore, the up-regulation of AT1-R in PVN may be mediated by MAPK signaling stimulation in PVN during hypertension. In this study, we found that PVN activities of p-p44/42 MAPK and p-p38 MAPK in 2K1C groups were enhanced more than those of SHAM groups. Eight weeks of aerobic ExT suppressed the PVN activities of p44/42 MAPK and p38 MAPK in 2K1C rats. Moreover, the existing research from our laboratory and other groups have reported that MAPK affects NF-κB activity as the upstream regulator in the PVN [26,47].

**Figure 9:**
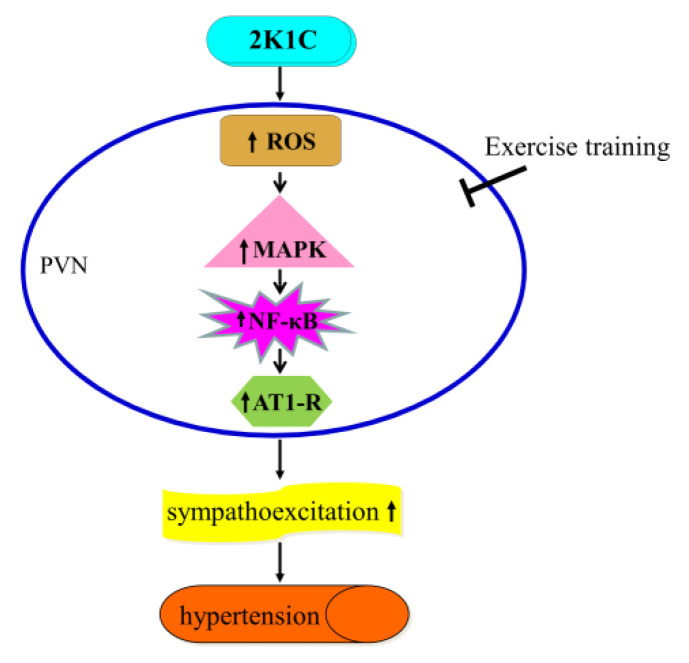
Graphical abstract of the mechanism of aerobic exercise training in ameliorating hypertension. ↑: Increased expression.

In summary, our data showed that the 2K1C surgery activated the PVN ROS/MAPK/NF-κB signaling overloading the AT-1R overexpression. This study revealed that aerobic ExT attenuated hypertension in 2K1C rats, and suppressed the ROS/MAPK/NF-κB/AT-1R pathway in the PVN of 2K1C hypertensive animals. Figure 9 illustrates the central mechanism of aerobic ExT attenuating hypertension. Aerobic ExT might be a potential treatment for hypertension through modulating the ROS/MAPK/NF-κB/AT-1R pathway in 2K1C hypertension.

## 5. Conclusions

Data from the current study supported that long-term aerobic ExT significantly inhibited the ROS/MAPK/NF-κB signaling in PVN and subsequently down-regulated AT-1R expressions, attenuated blood pressure of 2K1C rats, and consequently ameliorated the development of 2K1C-induced hypertension.

## Figures and Tables

**Table 1:** Primer sequences used for quantitative Real-time PCR.

Genes	Forward	Reverse
AT-1R	5′-CAACCTCCAGCAATCCTTTC-3′	5′-CCCAAATCCATACAGCCACT-3′
GAPDH	5′-AGACAGCCGCATCTTCTTGT-3′	5′-CTTGCCGTGGGTAGAGTCAT-3′

## Data Availability

All relevant data are within the manuscript and its Supporting Information files.

## Supplementary Materials

The following supporting information can be downloaded at: https://www.mdpi.com/article/10.3390/nu14193968/s1, Figure S1: ExT decreased p-p44/42 MAPK protein expression in 2K1C rats; Figure S2: ExT decreased p-p38 MAPK protein expression in 2K1C rats; Figure S3: ExT decreased AT-1R protein expression in 2K1C rats.

## Abbreviations

SNA: sympathetic nerve activity; PVN: paraventricular nucleus; RAS: renin-angiotensin system; ANG II: angiotensin II; AT-1R: angiotensin II type 1 receptor; RSNA: renal sympathetic nerve activity; ExT: exercise training; ROS: reactive oxygen species; MAPK: mitogen-activated protein kinase; JNK: c-Jun N-terminal kinase; p-: phosphorylated; NF-κB: nuclear factor-kappa B; SD: Sprague-Dawley; 2K1C: two-kidney, one- clamp; Sed: sedentary; SHAM: sham operation; SBP: systolic blood pressure; DHE: Dihydroethidium; NE: norepinephrine; RT-qPCR: quantitative real-time PCR.
