# Supplementary material for: Exercise Training Attenuates Hypertension via Suppressing ROS/MAPK/NF-κB/AT-1R Pathway in the Hypothalamic Paraventricular Nucleus
Source: Nutrients. 2022 Sep 24;14(19):3968. doi: 10.3390/nu14193968 (PMC9573547; doi:10.3390/nu14193968)
Supplement: Supplementary file 1 [file nutrients-14-03968-s001.zip › nutrients-1904232-supplementary.pdf]

Western blotting Gels:

Figure S1

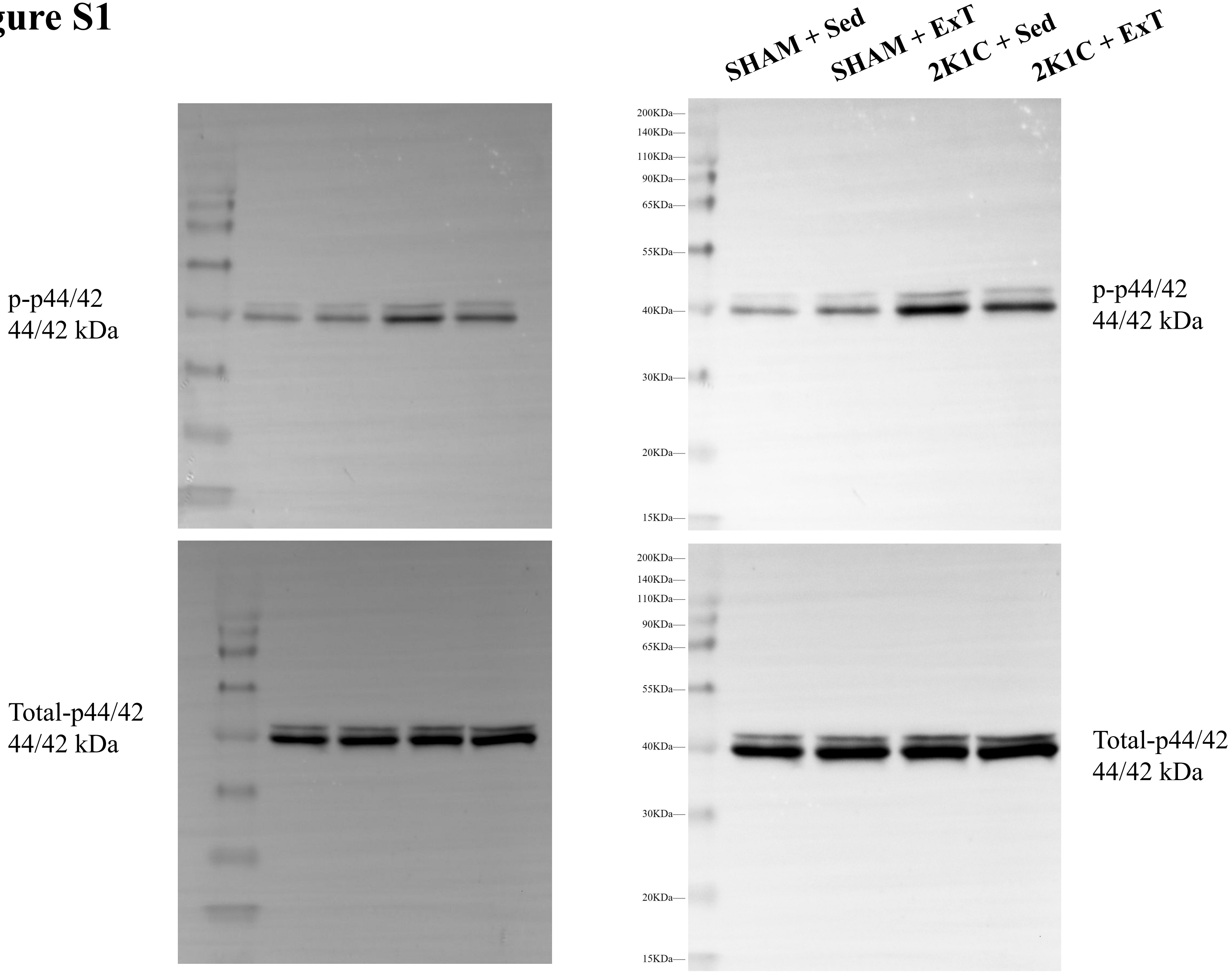

Figure S1: ExT decreased p-p44/42 MAPK protein expression in 2K1C rats.

Western blotting Gels:

Figure S2

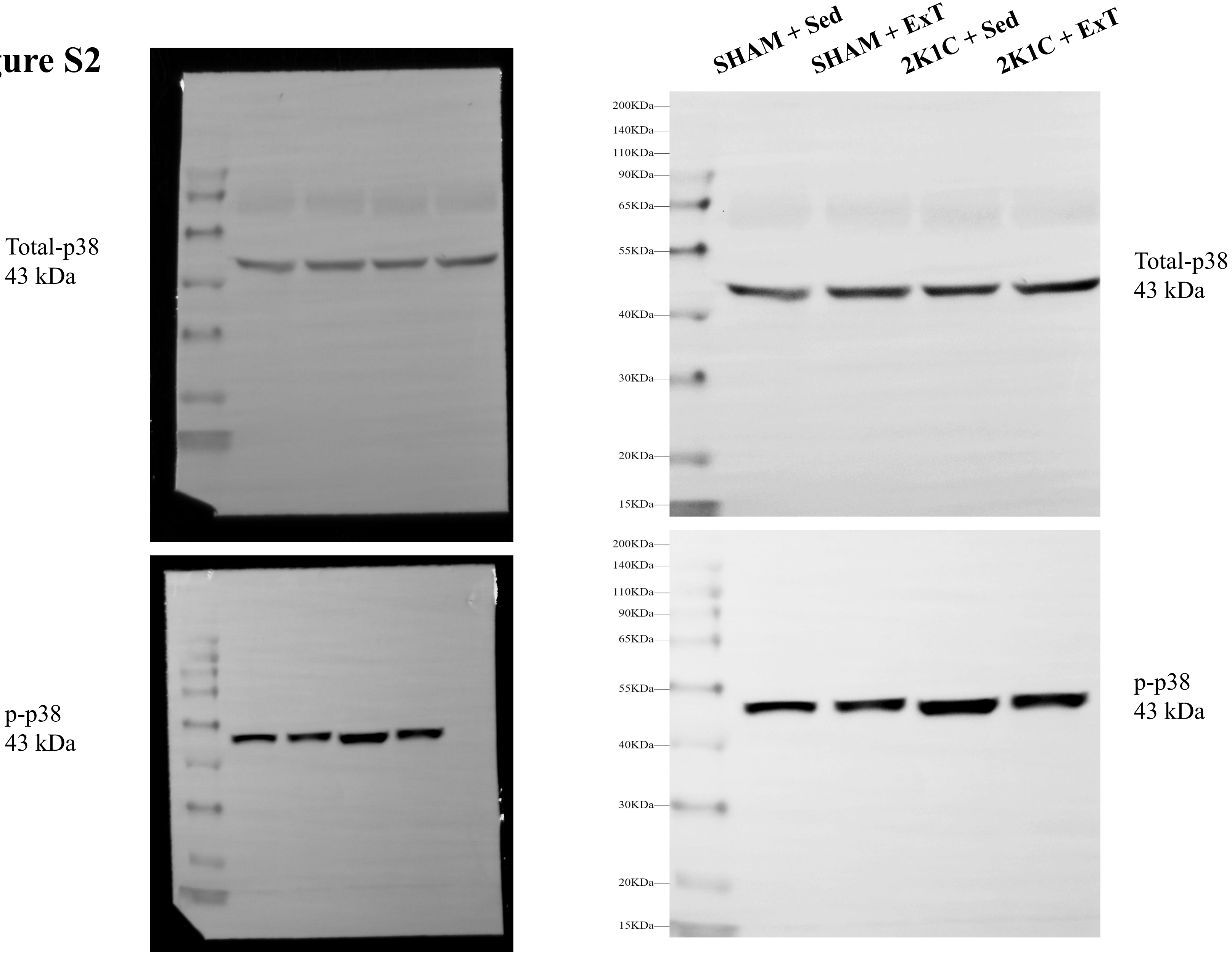

Figure S2: ExT decreased p-p38 MAPK protein expression in 2K1C rats.

Western blotting Gels:

Figure S3

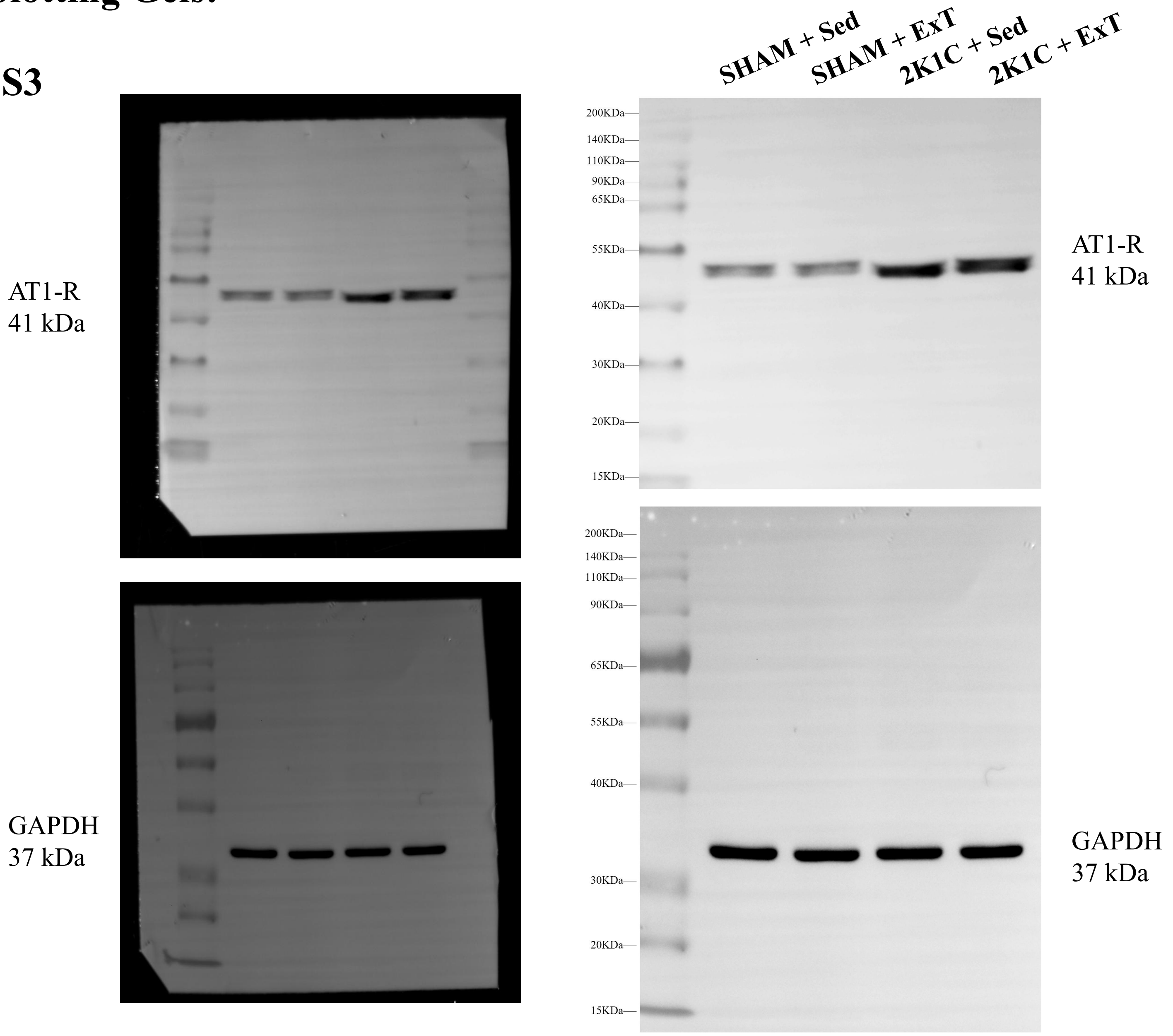

Figure S3: ExT decreased AT-1R protein expression in 2K1C rats.
